# MRI contrast agents and retention in the brain: review of contemporary knowledge and recommendations to the future

**DOI:** 10.1186/s13244-024-01763-z

**Published:** 2024-07-25

**Authors:** Josef Vymazal, Aaron M. Rulseh

**Affiliations:** Department of Radiology, Na Homolce Hospital, Roentgenova 2, Prague, 150 30 Czech Republic

**Keywords:** MRI contrast agents, Gadolinium, Relaxivity, Gadolinium retention, Ferritin

## Abstract

**Abstract:**

Gadolinium-based contrast agents (GBCA) were introduced with high expectations for favorable efficacy, low nephrotoxicity, and minimal allergic-like reactions. Nephrogenic systemic fibrosis and proven gadolinium retention in the body including the brain has led to the restriction of linear GBCAs and a more prudent approach regarding GBCA indication and dosing. In this review, we present the chemical, physical, and clinical aspects of this topic and aim to provide an equanimous and comprehensive summary of contemporary knowledge with a perspective of the future. In the first part of the review, we present various elements and compounds that may serve as MRI contrast agents. Several GBCAs are further discussed with consideration of their relaxivity, chelate structure, and stability. Gadolinium retention in the brain is explored including correlation with the presence of metalloprotein ferritin in the same regions where visible hyperintensity on unenhanced T1-weighted imaging occurs. Proven interaction between ferritin and gadolinium released from GBCAs is introduced and discussed, as well as the interaction of other elements with ferritin; and manganese in patients with impaired liver function or calcium in Fahr disease. We further present the concept that only high-molecular-weight forms of gadolinium can likely visibly change signal intensity on unenhanced T1-weighted imaging. Clinical data are also presented with respect to potential neurological manifestations originating from the deep-brain nuclei. Finally, new contrast agents with relatively high relaxivity and stability are introduced.

**Critical relevance statement:**

GBCA may accumulate in the brain, especially in ferritin-rich areas; however, no adverse neurological manifestations have been detected in relation to gadolinium retention.

**Key Points:**

- Gadolinium currently serves as the basis for MRI contrast agents used clinically.
- No adverse neurological manifestations have been detected in relation to gadolinium retention.
- Future contrast agents must advance chelate stability and relativity, facilitating lower doses.

**Graphical Abstract:**

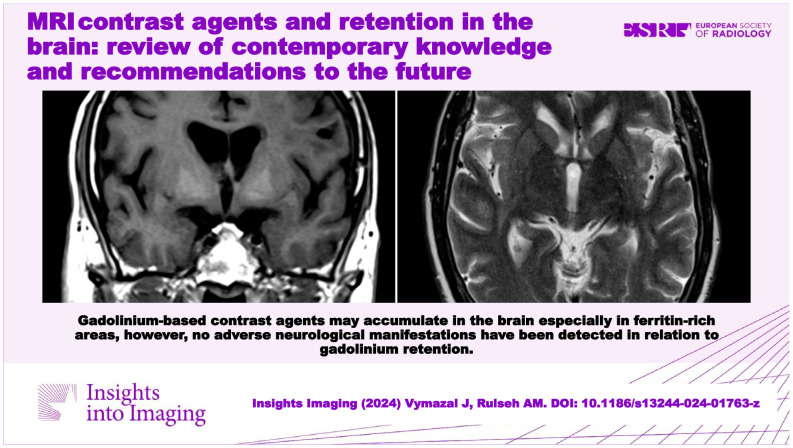

## Introduction

The emergence of any revolutionary method in medicine is usually associated with primary enthusiasm, followed by skepticism arising from unexpected side effects. The X-ray studios that arose shortly after Roentgen’s discovery were commercial attractions or parts of amusement parks. Although Clarence Dally, an assistant to Thomas Edison, was probably the first documented victim of X-ray radiation, there had been earlier reports about the possible side effects of X-rays; they were, however, mostly ignored [1, 2]. Currently, a significant source of population-level radiation exposure comes from computed tomography (CT) [3]. CT radiation doses exceed those from standard X-rays by an order of magnitude or more [4]. Thus, the emergence of magnetic resonance imaging (MRI) in the 1980s was accompanied by high expectations for a new technique that would provide optimal tissue contrast without harmful side effects. Presently, forty years after its introduction into clinical practice, there are no known health hazards from temporary exposure to the MRI environment [5].

Intravascular contrast agents used in MR imaging may significantly enhance the utility of the examination. The first MRI contrast agents, based on chelates of gadolinium (Gd), were introduced several years after the emergence of MRI with expectations for high efficacy and, contrary to iodinated contrast agents, low (nephro) toxicity and minimal allergic-like reactions. Despite a great number of gadolinium-based contrast agent (GBCA) applications, more than fifteen years passed before the first reports of systemic nephrogenic fibrosis (NSF) were published, and several more years to firmly relate NSF to GBCA application in patients with severely impaired renal function [6]. NSF has been shown to be related to GBCA chelate stability, and the restriction of GBCAs [7] with the least stable chelates in patients with impaired renal function led to a dramatic decrease in new NSF cases [8, 9]. In 2021, roughly 450 million GBCA applications were estimated since its introduction [10] and this number increases every year.

Restored confidence in the safety of GBCAs did not last long. In 2014, Kanda documented increased signal intensity in the dentate nucleus (DN) and globus pallidus (GP) following GBCA administration [11]. This phenomenon was recognized earlier, and was inaccurately attributed to multiple sclerosis [12] or brain irradiation [13]. Kanda was the first to attribute this increased signal intensity to previous GBCA administration [11, 14] and further observations followed (e.g., refs. [15–17]). Histochemical confirmation of residual Gd in dose-dependent quantities in the DN and GP, as well as other brain regions [18], has reinvigorated discussion regarding the safety of these contrast agents [19]. Gd deposition, mainly following the administration of simple linear GBCAs [18] (also primarily responsible for NSF), led to severe restrictions in the use of these GBCAs in the EU market to prevent any potential adverse outcomes that could be associated with Gd deposition in the brain [20]. It should be noted however, that relevant data regarding Gd deposition in various organs were available for a relatively long time before Kanda’s publication (e.g., [21]). The reason these studies documenting Gd deposition did not generate sufficient attention may be due to the fact that they did not emphasize Gd deposition in the human brain; a phenomenon that naturally raises concern.

In the following review, we present the chemical, physical, and clinical aspects of this important topic and aim to provide an equanimous and comprehensive summary of contemporary knowledge with a perspective to the future. We reference a number of quality publications concerning basic research, clinical MRI, and potential neurological manifestations, obtained from following the topic, as well as by searching the PubMed database with relevant combinations of keywords (gadolinium, gadolinium retention, brain, GP, DN, and ferritin).

## MR considerations

For a deeper understanding of the properties of different contrast agents, it is important to explain the differences between magnetization and relaxation. Magnetization is defined as the value of the magnetic moment of a given substance placed into an external magnetic field per given volume, and is expressed in Bohr magnetons [22]. Elements or compounds with high magnetization therefore change the local magnetic field and increase local field inhomogeneities compared to neighboring structures. As this happens at a very small scale, we speak about micro-inhomogeneities. As a result, T2* relaxation time is shortened, because contrary to T2 relaxation, no correction 180° pulse is applied in the T2* relaxation process. Thus, elements or compounds with high magnetization (e.g., dysprosium) are useful as “susceptibility contrast agents”. T2* relaxation shortening is not necessarily followed by T1 shortening; therefore, susceptibility agents are not used in postcontrast T1-weighted imaging.

**Fig. 1:**
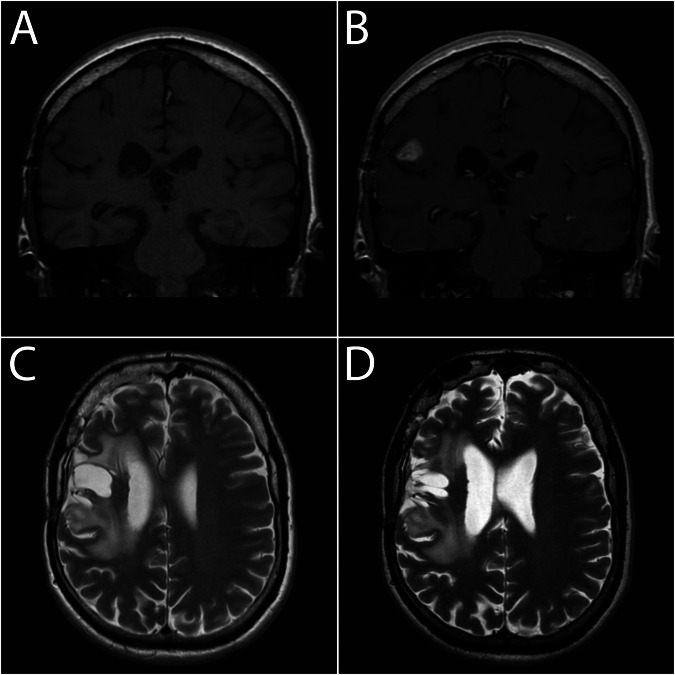
Effect of GBCA on T1- and T2-weighted images: **A** precontrast T1-weighted image, (**B**) postcontrast T1-weighted image with postcontrast enhancement, (**C**) precontrast T2-weighted image, and (**D**) postcontrast T2-weighted images. No signs of T2 postcontrast enhancement are present, although as seen in (**B**) the blood-brain barrier is impaired

Relaxation is the process in which spins release the energy received from a radiofrequency pulse applied at the Larmor frequency, which linearly increases with increasing external magnetic field strength [23]. Furthermore, relaxivity is defined as a property of a given substance to shorten T1 and T2 relaxation times. T1 shortening is always accompanied by the shortening of T2 relaxation, however, the opposite does not apply. T2 shortening in imaging with Gd-based contrast agents is generally not clinically significant and is used in T2* susceptibility imaging. Why is T2-weighted imaging insensitive to GBCAs when there is also T2 shortening? The answer is clear when we consider tissue T1 and T2 relaxation times. T1 tissue relaxation times are in the range of 1 s^−1^, while T2 relaxation times are in the range of roughly 10 s^−^^1^. An increase of, for example, 2 s^−^^1^ from GBCA application results in a change of 200% in T1 relaxation, while only roughly 20% in T2 relaxation. Therefore, the effect of standard GBCAs on T2-weighted images is negligible and mostly invisible to the naked eye (Fig. 1). This does not apply to postcontrast FLAIR due to the mild T1 effect induced by the long inversion time [24].

## Specific elements and molecules as MR contrast agents

A number of elements and molecules have been evaluated as potential MR contrast agents, and this remains an area of active research. Many elements and molecules exhibit unique properties in the MR environment, and thus may facilitate unique clinical applications; however, unique toxicities of potential contrast agents must also be considered. Below we detail some compounds that have been evaluated as MR contrast agents.

### Dysprosium

Dysprosium has a larger magnetic moment than Gd, however it is far less effective in shortening relaxation times (lower relaxivity) in comparison with Gd [25, 26] (Table 1). The toxicity of both metals is similar. Due to the large magnetic moment of dysprosium, a contrast agent called Sprodiamide was tested to serve as a potential MRI susceptibility contrast agent [27, 28]. However, it was never approved for human or veterinary use. Thus, it is important to emphasize that an element with a high magnetic moment (e.g., dysprosium) may not serve as an optimal contrast agent as relaxation is more important than magnetization.

**Table 1:** Relaxivity and magnetization of basic elements

Element	Magnetic moment (Bohr magnetons)	T1 relaxivity 1.5 T (s^−^^1^)
Dysprosium	10.60	0.55
Gadolinium	7.05	8.9
Manganese	3.94	5.2
Iron	3.22	7

### Manganese

Manganese is a non-lanthanide element capable of serving as an MRI contrast agent. Non-chelated manganese has a reasonably high T1 relaxivity of 5.2 s^−1^ at 1.5 T and body temperature, compared to non-chelated Gd with a value of 8.9 s^−^^1^. Non-chelated manganese shows very high T2 relaxivity with a value of ca 65 s^−^^1^ at 1.5 T and body temperature, due to contact interactions [25]. This quality is however annulled by chelation [25] and the only approved manganese-based MRI contrast agent, mangafodipir (Teslascan), used its effect on T1 relaxation [29, 30]. However, due to low sales, concerns over toxicity, and the emergence of liver-specific Gd-based MRI contrast agents, this contrast agent was withdrawn from the US market in 2003 and from the EU in 2010 [31].

Questions surrounding Gd retention have nevertheless reinvigorated attempts to use manganese as an MRI contrast agent. A potential manganese-containing MRI contrast agent called EVP-1001 [32] has completed phase II clinical trials for imaging myocardial viability, and an Mn-based nanoparticle formulation named SN132D with a T1 relaxivity (R1) of roughly 25 s^−^^1^ at 1.5 T and 37 °C is currently being tested for breast cancer imaging [33]. Contrary to organ-specific manganese contrast agents under development and described above, there is promising potential for a manganese-containing MRI contrast agent called Mn-PyC3A, with a stable chelate and comparable relaxivity and pharmacokinetics to extracellular fluid GBCAs [34].

### Iron

Iron oxide nanoparticles have been used as T2 contrast agents for decades [35]. Pioneering work in the development of these agents was done by Bulte et al [36] shortly after the emergence of the first MRI contrast agents. Some of these agents have reached approval for clinical imaging, e.g., ferucarbotran [37], and were intended primarily for liver imaging. The primary reason these agents have mostly disappeared from clinical imaging is likely that radiologists vastly prefer positive contrast agents on T1-weighted imaging. However, ultrasmall iron oxide nanoparticles (USPIO) with core sizes less than 10 nm can more effectively reduce T1 relaxation times and therefore work as T1 contrast agents, but at the expense of their T2 shortening capability. Due to the lack of nephrotoxicity and their biodegradability, they may find their way into clinical imaging [38]. An example may be ferumoxytol, approved for anemia treatment, which provides strong T1 and T2 relaxation shortening and has been used off-label as a promising contrast agent [39].

### Other molecules

Recently, some less common molecules have been tested as potential MRI contrast agents, e.g., hyperpolarized [^15^N_2_] urea [40] or hyperpolarized water [41] for perfusion imaging. Relaxivity and magnetization of basic elements with potential as MRI contrast agents are listed in Table 1. However, it is important to note that relaxivity values are presented in the free (not chelated) form and that chelation decreases (in some cases significantly) the final relaxivity of the compound.

## GBCA

More than twenty years ago, Peter Caravan called Gd, “an obscure lanthanide element buried in the middle of the periodic table” [42]. How did this metal named after Finnish “element hunter” Johan Gadolin [43] become commonplace in medical diagnostics? In the search for the most beneficial contrast agent for routine clinical practice, various lanthanides were studied and are still under consideration [44].

Practically all clinical MRI contrast agents in current use are based on chelates of Gd. This rare earth metal has become ubiquitous in the environment over the years, and can be found in trace amounts in drinking water, rivers, and lakes [25, 45, 46]. Gd is toxic in its free form, at least in part due to the similar ionic radius of Gd^3+^ and Ca^2+^, which leads to the blockage of voltage-gated calcium channels and the inhibition of some enzymes [47]. GBCAs are therefore synthesized in the form of chelates that eliminate toxicity when stable. A chelate is a compound formed by the bonding of a metal (in this case, Gd) with a ligand (molecule). According to the chemical structure of a chelate, GBCAs can be divided into three groups: simple linear, substituted linear, and macrocyclic (Table 2).

**Table 2:** A list of GBCAs and their relaxivities at 1.5 T and 3 T

GBCA type	Generic name	Ligand	Commercial name	T1 relaxivity at 1.5 T	T1 relaxivity at 3 T
Simple linear	Gadopentetate dimeglumine	DTPA	Magnevist	3.9	3.9
Simple linear	Gadodiamide	DTPA-BMA	Omniscan	4.5	3.9
Simple linear	Gadoversetamide	DTPA_BMEA	Optimark	4.4	4.2
Substituted linear	Gadobenate dimeglumine	BOPTA	MultiHance	6.2	5.4
Substituted linear	Gadoxetic acid	EOB-DTPA	Primovist	7.2	5.4
Substituted linear	Gadofosveset trisodium^a^		Vasovist, Ablavar	8.9	6.1
Macrocyclic	Gadoteric acid	DOTA	Dotarem, Clariscan	3.9	3.4
Macrocyclic	Gadoteridol	HP-DO3A	Prohance	4.3	3.4
Macrocyclic	Gadobutrol	BT-DO3A	Gadovist, PixxoScan	4.7	4.5
Macrocyclic	Gadopiclenol		Vueway, Elucirem	12.8	11.6

According to refs. [56–58]

^a^ Withdrawn from the market

Simple linear chelates have an open-chain structure. An example of such a compound is gadopentetate dimeglumine (Gd-DTPA; Magnevist; Bayer HealthCare, Berlin, Germany), which was the first GBCA approved for clinical use in 1988. The stability of simple linear chelates is lower compared to other chelates, therefore excess ligand was added to the agent [7, 48]. The highest amount of excess ligand was given to gadodiamide (Omniscan; GE Healthcare, Milwaukee, WI; 12 mg/mL) and to gadoversetamide (Optimark; Liebel-Flarsheim Company LLC, Raleigh, NC; 28.4 mg/mL) [49]. The addition of excess ligand was driven by the idea that it would eventually capture free Gd released from the relatively unstable chelate. This likely did occur to some extent as other elements such as zinc or iron are attracted to the ligand/chelate in a process called transmetallation. Thus, the addition of free excess ligands did not prevent these contrast agents from playing an active role in the emergence of NSF in patients with severely impaired renal function. However, in a systematic review, linear non-ionic contrast agents were associated with the lowest incidence of allergic-like reactions from all contrast agents [50].

Substituted linear contrast agents are open-chain chelates that additionally contain an aromatic side chain that interacts with plasma proteins, thereby increasing the relaxivity of these agents by slowing the correlation time of the molecule [51]. Correlation time is the time required for a molecule to rotate by approximately 57°. Smaller molecules rotate faster and thus their correlation time is shorter. Adding an aromatic chain increases the size of the molecule and therefore decreases its correlation time, and decreased correlation time to the range of the Larmor frequency increases T1 relaxivity [52].

**Fig. 2:**
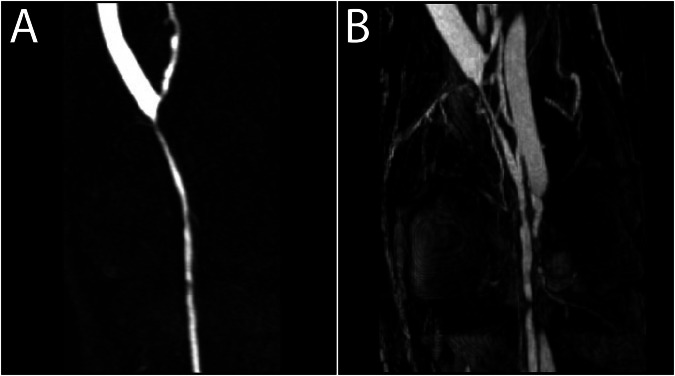
The effect of blood-pool GBCA Vasovist. **A** First-pass contrast-enhanced MR angiography showing probable stenoses of the popliteal artery. **B** Steady-state postcontrast image with a higher resolution and venous contamination. However, the degree of stenoses is better displayed

Increased relaxivity was the primary motivation for developing these GBCAs as described above. However, zero incidence of NSF after the application of gadobenate dimeglumine (Multihance; Bracco, Milan, Italy) [53, 54] or gadoxetate disodium (Primovist/Eovist; Bayer HealthCare, Berlin, Germany) [55] and the corresponding higher kinetic inertness of these agents led to the recognition that adding an aromatic chain also increased their stability. An explanation of this phenomenon lies in the concept of π-interactions [56]. No excess ligand is added to gadobenate dimeglumine (MultiHance) and macrocyclic gadoterate meglumine (Dotarem; Guerbet, Villepinte, France) (Table 2). A similar chelate structure with an aromatic side chain was present in the promising contrast agent gadofosveset trisodium (Ablavar/Vasovist; Lantheus Medical, North Billerica, MA), used primarily for “intravascular imaging” [57] and enabling steady-state imaging (Fig. 2), and which is unfortunately out of the market. Conversely, the interaction of GBCAs with a plasma protein was associated with a higher incidence of allergic-like reactions [50].

In macrocyclic agents, Gd^3+^ is “caged” in a cavity of the ligand [7]. Common sense suggests that these agents should be more stable compared to an “open-chain” structure; however, this is not necessarily true. Chemistry is complex and some of the above-mentioned substituted linear contrast agents may be more stable than macrocyclic agents. GBCAs with macrocyclic chelates used in clinical imaging include gadoterate meglumine (Dotarem), gadobutrol (Gadovist; Bayer HealthCare, Berlin, Germany; the only agent provided in a 1-M concentration), and gadoteridol (ProHance; Bracco, Milan, Italy). A promising new macrocyclic contrast agent, gadopiclenol (Bracco, Milan, Italy; Guerbet, Villepinte, France), will be described at the end of this review. Macrocyclic structure and ionicity have been associated with a higher risk of allergic-like reactions compared to non-ionic linear GBCAs [50]. A list of GBCAs and their relaxivities at 1.5 T and 3 T is provided in Table 2 [58–60].

### GBCA stability

Chelate stability is a crucial factor in the safety of any GBCA, especially from the point of view of Gd deposition in the brain and other organs, and with respect to potential clinical consequences. There have been discussions as to whether the thermodynamic stability of the chelate or its kinetic lability is the key factor in the stability of the molecule. Published data [61] have demonstrated that kinetic lability is the key factor in GBCA stability. Neburkova et al [61] showed that thermodynamic stability is comparable among clinically-used GBCAs, as well as in an experimental ethylenediaminetetraacetic acid (EDTA) Gd chelate (which is highly unstable), while Gd releases corresponding to the kinetic lability of the compound.

### Gadolinium retention in the body

Toxicity, as well as long-term retention of Gd in the reticuloendothelial system of the liver, was documented in 1981, before the emergence of MRI, when rare earth metals were studied as potential CT contrast agents [62]. In 1984, the Gd-DTPA chelate (Gd-DTPA; Magnevist), which later became the first clinically-approved GBCA, was tested by Weinmann et al [46]. They analyzed the toxicity and elimination from the body of Gd chloride, Gd-EDTA and Gd-DTPA. Gd-DTPA, having a stronger chelating agent compared to EDTA [63], provided the best results and the authors concluded that, “no evidence of dissociation of the Gd ion from the DTPA ligand was detected in vivo.” However, the total recovery of Gd-DTPA in rats was reported by the authors as 97.5%, with 0.08% retained in the liver, 0.01% in the spleen, 0.1% in the kidney, and 0.21% in the “remaining body”.

Thus, the accumulation of Gd in various organs was known already before the era of NSF and the later observation of Gd retention in the brain. However, Gd accumulation did not attract much attention and it was not expected that Gd can cross the intact blood-brain barrier [46]. We will not focus on NSF in the present review; many studies including a high number of reviews have been published on this topic and due to the restrictive approach in the application of linear GBCAs in patients with (severely) impaired renal function, NSF has been practically eliminated [9]. In the following text, we will focus on Gd deposition in the brain, especially in the deep-brain nuclei [46].

### Gadolinium retention in the brain

**Fig. 3:**
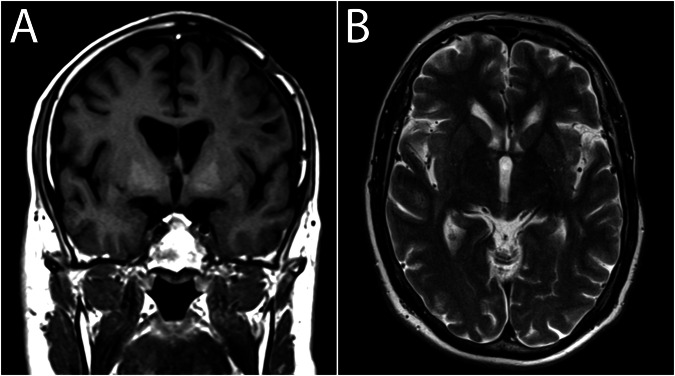
The effect of Gd–ferritin interactions. **A** non-contrast T1-weighted image displays visibly increased signal intensity in the globi pallidi. **B** T2-weighted image shows physiological hypointensity in the globi pallidi. Additional T2 shortening due to the Gd–ferritin interactions cannot be appreciated by the naked eye

The relationship between increased signal intensity in the DN and GP on native T1-weighted images and Gd was first made in 2014 [11]. These deep-brain nuclei are involved in motor control and a number of other functions, as described below. Little attention however was devoted as to why is Gd deposited in these nuclei, and also whether detected quantities of Gd can cause visible signal intensity increases on T1-weighted images. Another question that arises is if there is also an effect on T2 relaxation. This is an obvious consideration as selective T1 shortening due to (para)magnetic effects in reality does not exist. The converse is not true, there may be selective T2 shortening due to magnetic field inhomogeneity and consequent proton dephasing, e.g., due to the presence of deoxyhemoglobin. In this case, only the T2 effect applies because the proton–electron dipole–dipole interaction that would be responsible for the shortening of T1 relaxation is not present [64]. However, little attention has been given to the question of whether T2 shortening (possibly not apparent qualitatively) follows the T1 shortening visible as hyperintensity on non-enhanced T1-weighted images (Fig. 3).

What is common for the deep-brain nuclei and the presence of Gd? Deep-brain nuclei are known to accumulate ferritin and its concentration increases with age [65]. Ferritin is a metalloprotein consisting of a protein shell and core that can accumulate up to 4500 atoms of iron. The “loading factor” is however usually lower in vivo [66, 67]. The highest accumulation of ferritin-bound iron is in the GP, followed by other extrapyramidal centers such as the red nucleus, substantia nigra, putamen, and DN [65]. Ferritin is therefore responsible for low signal intensity on T2- or T2*-weighted images in the GP, and to a lesser extent other deep-brain nuclei. This effect is linearly dependent on the external magnetic field strength and thus more prominent at 3 T in comparison to 1.5 T [66].

Metalloproteins, however, including ferritin, can attract various metals. In 2011, Zhang [68] showed that gadodiamide (Omniscan) binds to apoferritin in vitro resulting in shortening of T1 relaxation time. In 2020, Neburkova et al [61] reported the shortening of both T1 and T2 relaxation times in vitro by Gd–ferritin complexes, by incubation of ferritin with various GBCAs [61]. It was proven that simple linear GBCAs such as gadodiamide (Omniscan) release significantly more Gd that consequently binds to ferritin compared to macrocyclic agents. However, an interesting phenomenon was also shown in substituted linear GBCAs; gadobenate meglumine (MultiHance) and gadoxetate disodium (Primovist) released less Gd than macrocyclic gadobutrol (Gadovist). The relationship between GBCAs and iron may be even more complex. In 2011, Ghio et al showed in vitro that Gd impacts cell iron homeostasis, affecting the import, storage, and biological effects of iron [69].

Mass spectrometry and electron microscopy studies in human subjects have proven the presence of Gd in the brains of subjects exposed to GBCAs, including subjects without intracranial pathology, while no Gd was detected in the brains of subjects not exposed to GBCAs [18, 70]. From the regions evaluated, the highest amount of Gd was found in the GP and dentate nuclei. While most Gd was found clustered in the endothelial wall, up to 42% was reported to have crossed the blood-brain barrier to deposit in the neuronal interstitium. Notably, no gross histological differences were noted in these subjects in comparison to controls. It is a question as to what extent the Gd clusters identified may be water-soluble. If insoluble in water, they would only selectively shorten T2* relaxation, similar to hemosiderin, and should not have any effect on T1 relaxation as spin–spin dipole interactions would not occur [71].

Thus, based on theoretical assumptions and real-world experiments, increased signal intensity in the GP and DN after GBCA administration is likely predominantly due to Gd–ferritin (macromolecular) interaction. This assumption does not exclude the presence of Gd in other forms and locations, however, its effect on the T1-weighted signal is likely predominantly due to the interaction of Gd with a macromolecule [61, 72].

### Relation between experimental and imaging data

With deeper insight into the issue of hyperintensity in the GP and DN on non-contrast T1-weighted images, a question arises, whether the detected concentrations of Gd in these nuclei are able to cause this signal change. McDonald et al [18] measured Gd concentrations in the human brain and presented MR images showing hyperintensity on non-contrast T1-weighted images in the GP (17.2 µg/g) and DN (58.8 µg/g). The measured concentrations in the pons (1.7 µg/g) and thalamus (2.3 µg/g) were lower by an order of magnitude. It is of interest that increased signal intensity was also observed in the pulvinar of the thalamus, a region of high iron concentration [73]. Another study in rat and human specimens also provides evidence that Gd retention is greatest in brain regions that exhibit iron (ferritin) enrichment [74].

In the same manner, Frenzel et al suggested [72] that insoluble low quantities of low-molecular forms of Gd cannot shorten T1 relaxation in such a manner that a visible increase in signal intensity would follow. They hypothesized that low quantities of Gd may be attached to a macromolecule larger than 250–300 Da, in order to cause a visible increase in signal intensity. Another study has shown that Gd retention corresponds with iron-rich areas [75], and regions rich in ferritin [67]. Recent proof of Gd–ferritin interactions perfectly matches this theory [61].

**Fig. 4:**
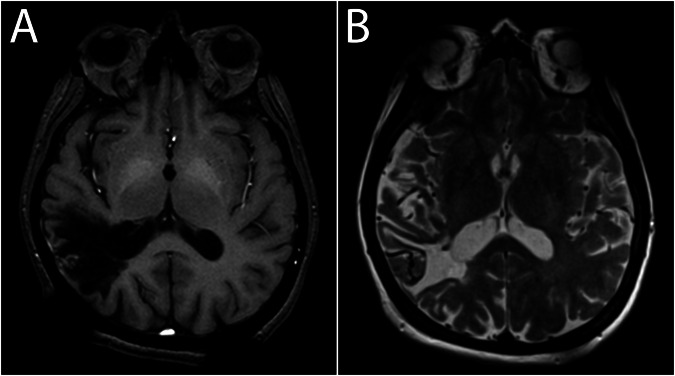
Hyperintensity in the GP in a patient suffering from liver cirrhosis. **A** non-contrast T1-weighted image displays symmetric hyperintensities in the globi pallidi. **B** T2-weighted image shows expected hypointensity in the globi pallidi. Additional T2 shortening due to the present T1 shortening cannot be appreciated by the naked eye. The peritrigonal lesion on the right side is a posthemorrhagic pseudocyst due to arteriovenous malformation

**Fig. 5:**
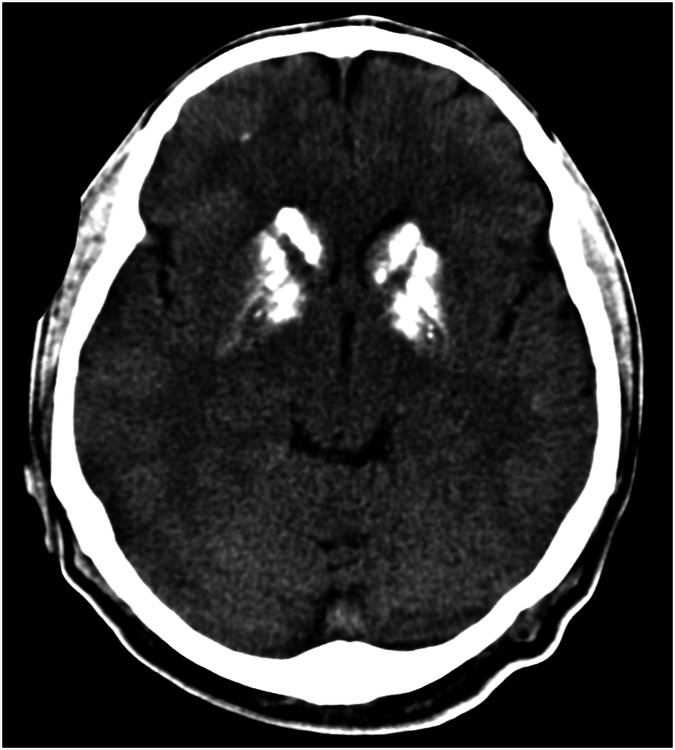
Fahr disease. CT image displays calcifications in regions rich in ferritin

Increased signal intensity in the deep-brain nuclei on non-contrast T1-weighted images is not always caused by Gd deposition. In 1996, Vymazal et al reported increased signal intensity in the GP in patients suffering from hepatic cirrhosis [76] (Fig. 4). This phenomenon is likely caused by the accumulation of paramagnetic manganese and is not specific to cirrhosis, rather situations where nutrients bypass the portal vein. Visible T1 shortening was followed by shortening of T2 relaxation times, detectable however only by relaxometry. It is of interest that after successful liver transplantation, the signal intensity in the GP returns to normal values [77]. In Fahr disease, calcium is accumulated in the GP, caudate, DN, and regions rich in ferritin, and it has been shown that elevated intracellular calcium increases ferritin expression (Fig. 5) [78]. Thus ferritin, similar to other metalloproteins, is able to interact with different elements including aluminum, manganese, calcium, and Gd. The common denominator in these diseases is therefore, with high probability, the formation of ferritin complexes with other metals or elements other than iron.

### Neurological manifestations of gadolinium deposition in the brain

Proven Gd deposition in various organs and tissues did not initially elicit much attention as discussed above, and the practical eradication of NSF further calmed the situation. The realization that Gd also deposits in the brain has however reinvigorated discussion about GBCA safety. Major sites of Gd deposition in the brain are, as mentioned, the deep-brain nuclei, especially the DN and GP. Therefore, if any clinical manifestations are to be expected, they should primarily originate from these nuclei.

The role of the GP and DN is, however, rather complex and is not limited to the control of movement. Thus, lesions of the GP may cause not only movement disorders (dystonia or parkinsonism) but also less intuitive manifestations such as apathy, depression, psychic akinesia, or micrographia [79]. Thorough neurological examination and neuropsychological testing are therefore critical. Lesions of the DN may cause dysarthria, ataxia, and cognitive manifestations such as working memory deficiencies or decreased cognitive flexibility [80].

No prospective studies have been published due to restrictions in the use of simple linear GBCAs that result in the greatest Gd accumulation in the deep-brain nuclei; it would be ethically unsound to conduct such a study. Retrospective data are also scarce. Welk et al [81] statistically compared roughly 150,000 patients with no Gd exposure with approximately 100,000 patients with a history of at least one GBCA application; no significant association between Gd exposure and parkinsonism was found.

It is practically impossible to find a population of patients who received a large amount of GBCAs over a longer period of time without significant neurological disease. Theoretically, such a study could include female subjects at high risk of breast cancer who repeatedly underwent an MRI examination with GBCA application. However, no such study has yet been published.

Vymazal et al [82] published a study on four subjects who received between 561–915 mL of various GBCAs, linear and macrocyclic, over a period of 14 years. These patients were treated and followed for glioblastoma and therefore they had very frequent MRI follow up. A thorough neurological examination, focused on the detection of even subtle signs of movement disorders, natural history, and neuroprotection in Parkinson plus syndromes—Parkinson plus scale, was negative in all patients. The neuropsychological examination did not reveal any aberrant findings beyond those that could be ascribed to each patient’s premorbid intellectual capacity, to the location and progression of GBM, and to the therapeutic regimens undertaken.

Thus, is the detectable signal intensity increase on T1-weighted non-contrast images, made possible by interaction of small amounts of Gd with a macromolecule only an epiphenomenon with no clinical significance? So far there is no clear evidence that it should be considered otherwise. Does the signal intensity later decrease or even return to normal values after the exposure of Gd has been stopped, as in the case of manganese accumulation in patients after successful liver transplantation? Indeed, a study in rats showed partial Gd clearance from the brain twenty weeks after repeated gadodiamide administration was ceased [83].

## Future MR contrast agents

In the future, several conditions for contrast agents should be fulfilled, regardless of whether they contain Gd or not. These conditions were not a priority in the past, especially before the era of NSF and the discovery of Gd retention in the brain. Future contrast agents will need to be stable, in the case of GBCAs, they should be of macrocyclic or substituted linear structure, as it was proven that substituted linear agents may be more stable than some macrocyclic agents [61]. New contrast agents will need to have high relaxivity in order to achieve optimal effect at the lowest possible dose. This ALARA concept was known but sometimes ignored to achieve high diagnostic performance with standard- or low-relaxivity agents, when double or even triple doses were recommended (e.g., in imaging brain metastases). The argument that a double or a triple dose probably does not do too much harm to a patient with brain metastases and that diagnostic yield is more important is not fully relevant. It has been stated recently that Gd load not only to the patient but also to the environment should be considered [84].

One promising molecule is gadopiclenol [85]), which was recently approved by the Food and Drug Administration and by the European Medicines Evaluation Agency. This contrast agent is macrocyclic and has a very high relaxivity of ca 12 at both 1.5 T and 3 T, and a kinetic stability of more than 20 days. Why does this molecule have such properties? Contrary to all other GBCAs, gadopiclenol has two water molecules in the inner coordination sphere of the molecule [86], thus increasing its relaxivity. Side chains are responsible for both increased relaxivity by decreasing the tumbling time and also for increased stability, similar to substituted linear GBCAs.

Other macrocyclic, high relaxivity agents are also being developed, notably gadoquatrane and gadoPlus [87]. However, their approval by regulative authorities is expected in the range of several years. Metal-free MR contrast agents are promising, but their development will probably take a long time. Another important field is “molecular contrast agents” which provide additional information compared to what can be achieved with standard MR imaging in terms of targeting specific proteins, cell types, or biological processes [88]. These contrast agents have the capacity to increase the sensitivity and specificity of MR imaging in a one-stop imaging process. A detailed description of these promising agents is, however, beyond the scope of this review.

In summary, a number of metals have been investigated in the search for MR contrast agents. Gadolinium currently serves as the basis for all MR contrast agents used clinically. GBCA stability has proven its importance, first with the discovery of NSF, and later with the observance of Gd retention in the brain. Although NSF has practically been eliminated, Gd retention remains a contemporary concern. However, no adverse neurological or psychological manifestations have been detected in subjects with documented Gd retention in the brain. Gd precipitates have been detected in the endothelium and across the intact blood-brain barrier in healthy subjects exposed to GBCAs, however, MRI signal changes are likely due to complexes formed with a macromolecule such as ferritin. In either case, Gd would intuitively be sequestered from interaction with the cellular environment. Nevertheless, the processes behind Gd retention have not yet been fully elucidated, and justifiable concerns remain; this remains a limitation in the interpretation of potential sequelae, as well as the present review. Other limitations may include the involvement of other brain areas that have not been fully investigated (e.g., putamen and caudate nucleus). Some discrete neurological symptomatology may appear after a long time interval, and is a further potential limitation. Future gadolinium-based MR contrast agents should be stable (preferably macrocyclic) chelates, and have high relaxivity that enables the detection of discrete pathological lesions (e.g., brain metastases or acute demyelinating plaques) with a minimal effective dose, thus minimizing gadolinium retention in the body and protecting the environment.

## Abbreviations

CT: Computed tomography
DN: Dentate nucleus
EDTA: Ethylenediaminetetraacetic acid
GBCA: Gadolinium-based contrast agent
Gd-DTPA: Gadopentetate dimeglumine
GP: Globus pallidus
MRI: Magnetic resonance imaging
NSF: Nephrogenic systemic fibrosis
